# The effect of home gardening on vegetable and fruit consumption: a pre-post intervention study in Northeast Hungary

**DOI:** 10.1186/s41043-025-01217-2

**Published:** 2026-01-27

**Authors:** Anita Simon, Helga Bárdos

**Affiliations:** 1 Doctoral School of Health Sciences, University of Debrecen, Debrecen, Hungary; 2 Department of Public Health and Epidemiology, Faculty of Medicine, University of Debrecen, Debrecen, Hungary

**Keywords:** Home gardening, Fruit and vegetable intake, Rural health, Public health intervention, Nutrition education

## Abstract

**Background:**

Diet low in vegetables and fruits is one of the leading dietary risk factors for non-communicable diseases. In Hungary, the average consumption of vegetables and fruits is less than the recommended daily amount. Home gardening is a feasible option in rural areas and could provide a sustainable way to increase daily vegetable and fruit intake. This study evaluated the effect of a home gardening intervention on vegetable and fruit consumption in two rural settlements in Hungary.

**Methods:**

A pre-post interventional study was conducted between May and September 2022 with the participation of 50 adults. The intervention included education on gardening, nutrition and healthy cooking techniques. The participants were provided with seeds and seedlings, and an information booklet. Before and after the intervention a questionnaire was used to collect information on sociodemographic data, vegetable and fruit consumption, knowledge of healthy diet, physical activity, weight and height, and wellbeing. The differences between vegetable and fruit consumption, and other variables measured before and after the intervention were tested using statistical tests.

**Results:**

The consumption of fruits and vegetables increased from a median of 1.6 (IQR 0.8–2.7) servings to 5.5 (IQR 3.9–7.6) servings per day (*p* < 0.001). The proportion of participants meeting the recommended intake of at least 5 servings per day increased from 10% to 60% (*p* < 0.001). Nutrition knowledge and physical activity slightly improved (*p* < 0.001), while BMI did not significantly change.

**Conclusion:**

The complex home gardening intervention had a positive effect on the vegetable and fruit intake of the study participants. Home gardening is potentially a feasible way of promoting healthier eating habits in rural communities.

## Introduction

High intake of fruits and vegetables is widely recognized as being beneficial in the prevention of chronic diseases, including cardiovascular diseases, cancer, diabetes, and Alzheimer’s disease [1, 2]. In 2017, 11 million deaths and 255 million disability-adjusted life years (DALYs) were attributed to dietary risk factors globally. The most significant contributors among these were high sodium intake (3 million deaths), low whole grain intake (3 million deaths), and low fruit intake (2 million deaths) [3].

The World Health Organization (WHO) recommends a daily intake of 400–500 g vegetables and fruits (VFs), ideally distributed across five meals per day [4]. According to the Global Burden of Disease Study (2017), the optimal mean daily intake is 250 (200–300) grams for fruits and 360 (290–430) grams for vegetables [3]. Despite these recommendations, average intakes remain low in many countries. Importantly, fruits and vegetables, although often studied together, differ in nutritional composition. Fruits are generally higher in sugar and calories but contain less dietary fiber compared to vegetables. This has particular relevance for individuals with diabetes or insulin resistance, as certain fruits (e.g., grapes, oranges, strawberries, and melons) can elicit stronger glycemic responses [5].

Vegetables, on the other hand, tend to be relatively high in water and dietary fiber but low in calories, which contributes to early satiety and reduced overall energy intake. Additionally, dietary fiber fermentation supports gut microbiota, promotes bowel regularity, binds toxins, and accelerates intestinal transit [6]. Adequate dietary fiber intake has been associated with a reduced risk of colorectal cancer [7]. Fruits and vegetables are also essential sources of vitamins, minerals, and phytochemicals with antioxidant, phytoestrogenic, and anti-inflammatory properties [6, 8].

Adopting a healthy diet is complex and influenced by variety of factors, including personal motivation, cultural norms, education, socioeconomic status, and geographic location [9]. National surveys in Hungary revealed alarmingly low consumption of vegetables and fruits. A household budget survey, which calculated food intake based on households’ expenditure, revealed in 2020 that the average daily intake was 138 g for vegetables and 128 g for fruits [10]. The 2019 Hungarian Nutrition Survey, as part of the European Health Interview Survey, was a dietary survey involving a 3-day food diary completed by a sample of participants. The average daily intake observed was 339 g, comprising 190 g of vegetables and 149 g of fruit [11].

Consumption varied by age and settlement type, with older adults and urban residents typically consuming more fruits and vegetables than younger individuals and rural inhabitants.

Home gardening has been proposed as a sustainable and accessible strategy to improve vegetable and fruit intake and address public health challenges such as malnutrition, food insecurity, and poor dietary quality. In developing countries, homestead food production programs targeting women and children have been effective in reducing undernutrition [12–14]. In high-income countries, gardening interventions have shown promise for specific populations, including cancer survivors, by improving dietary intake and overall well-being [15–17].

The aim of this study was to evaluate the impact of a structured home gardening intervention on vegetable and fruit intake, nutrition knowledge, physical activity, and other health-related outcomes among rural residents in Northeast Hungary.

## Methods

### Study participants

Fifty adults were recruited from two rural settlements in Northeast Hungary. Eligibility criteria included being 18 years of age or older and having access to a cultivable garden. Participants were recruited through online advertisements in local newspapers, and social media targeting residents’ groups. Printed flyers were placed in mailboxes, and posters were displayed in public places, such as post offices, schools, shops, and municipal buildings.

The required sample size was determined using the MedCalc software [18]. We calculated the required sample size for comparison of two related proportions as analyzed with the McNemar test. We assumed 2% of participants shift from positive to negative (i.e. from meeting the recommended vegetable and fruit intake to not meeting it), and 25% to shift from negative to positive. We selected 0.05 for α-level and 0.20 for β-level. The minimum required number of pairs (sample size) was calculated to equal 38.

### Study design

This study employed a pre-post interventional design and measured vegetable and fruit consumption before and after a home gardening intervention program in the same participants. The intervention was conducted from late May to early September 2022, a period of 3.5 months.

### Intervention

The intervention included three structured group sessions each 1.5–2 h long, which were delivered across the study period. The first session, held in late May, focused on basic gardening techniques including soil preparation, sowing, planting, irrigation, and pest control. The participants received seedlings (pepper, tomato, and zucchini) and seeds (radish and lettuce). Each participant was also provided with a 30-page booklet covering gardening techniques, nutritional guidance, and healthy cooking methods. The second session, held in mid-July, covered the principles of balanced nutrition and practical culinary techniques, such as healthy food preparation methods like steaming, boiling and roasting with limited oil. It also covered the basics of food storage and preservation. This session emphasized the macronutrient and micronutrient composition of food and healthy food preparation methods. The third session, held in early September, focused on preparing the winter garden, sharing experiences, and evaluating the program. Throughout the program, communication was maintained via a dedicated Facebook group and email summaries were sent after each session. There was no loss to follow-up during the study period.

### Data collection

Data were collected at two points, before the intervention in May 2022 and after the intervention in September 2022. Participants either completed a structured questionnaire on paper or digitally. The questionnaire assessed their demographics, gardening practice, vegetable and fruit consumption, knowledge about healthy nutrition, physical activity, weight and height, presence of long standing health problem and general activity limitation, and well-being.

## Measured variables

### Demographics

The questionnaire collected data on participants’ age, sex, employment status, educational level, marital status, household composition, perceived financial situation, and settlement location.

### Gardening practices

We asked the participants if they had any prior experience of gardening. We also asked them about their gardening location, the size of their garden, and the number of plant types they typically cultivate (if applicable).

### Vegetable and fruit intake

Vegetable and fruit consumption was measured by asking about frequency and quantity. The frequency options were: “more than once a day,” “once a day,” “six times a week,” “five times a week,” “four times a week,” “three times a week,” “two times a week,” “once a week,” and “less than once a week.” The quantity option was the average number of servings consumed at one time. A serving size was explained. Vegetable and fruit consumption was calculated as the number of daily servings. The number of daily servings according to the WHO recommendation [4] was used to define and calculate the “Meeting the recommendation” variable. The recommended daily intake of vegetables and fruits has been defined as five portions, which means three portions of vegetables and two portions of fruit per day.

### Healthy nutrition knowledge

Five simple statements related to current scientific opinions on diet and health were formulated to assess participants’ knowledge of healthy nutrition. The participants indicated their agreement with the true statements on a 5-point Likert scale (1 = strongly disagree, 2 = disagree, 3 = neither agree nor disagree, 4 = agree, 5 = strongly agree). The statements were as follows: (1) Adequate dietary fiber consumption (whole grain cereals, vegetables, fruits) reduces the risk of developing cardiovascular diseases; (2) At least 5 servings of vegetables and fruits should be consumed daily; (3) A healthy diet consists of 5 meals a day; (4) High salt intake can cause high blood pressure and heart disease; (5) Vegetable oil consumption is healthier than consuming animal fats. The average score reflected the agreement with the true statements, which were used to indicate a “healthy nutrition knowledge” on a 5-point scale. We considered a score of 1 to indicate low (bad) knowledge, and a score of 5 to indicate high (good) knowledge.

### Physical activity

The European Health Interview Survey–Physical Activity Questionnaire (EHIS-PAQ) as used to assess moderate to vigorous physical activity (MVPA) related to work, transport, and leisure time. The participants were classified as physically active if they engaged in at least 150 min per week of aerobic physical activity or equivalent work-related physical activity [19].

### BMI, presence of health problems

The participants reported their weight and height, which were used to calculate BMI in kg/m^2^. The presence of chronic health problems was asked, ‘Do you have any longstanding illness or health problem? (By longstanding, I mean illnesses or health problems that have lasted, or are expected to last, for 6 months or more.)’ Options were yes or no. General activity limitations due to health problems were asked ‘For at least the past 6 months, to what extent have you been limited because of a health problem in activities people usually do?’ The options were severely limited, limited but not severely limited, and not limited at all. The responses were categorized as either ‘general activity limitation is present’ (options ‘severely limited’ and ‘limited but not severely’) or ‘general activity limitation is not present’ (option ‘not limited at all’).

### Well-being

The participants were also asked, ‘Overall, how satisfied are you with your life these days?’ They had to rate how satisfied they were with their lives on a scale from 1 to 10. Larger values indicate a greater degree of satisfaction with life.

### Statistical analysis

Data were analyzed using IBM SPSS Statistics Version 25. The distribution of the data is presented as numbers and percentages for categorical variables, and as means (standard deviations) or medians (interquartile ranges) for continuous variables. We statistically tested differences in vegetable and fruit consumption, as well as other variables, before and after the intervention. Within group comparisons were made by using the Wilcoxon signed rank test for non-normally distributed variables (fruit and vegetable consumption, nutrition knowledge score and well-being), the paired t-test for normally distributed variables (BMI) and the McNemar test for categorical variables (meeting recommendations and physical activity). Differences in the changes in the measured variables between participants by previous gardening practice were statistically analyzed using Mann-Whitney U test for non-normally distributed variables (vegetable and fruit consumption, nutrition knowledge score, and well-being); the independent samples t-test for normally distributed variables (BMI); and Fisher’s exact test for categorical variables (meet the recommendations, physical activity).

## Results

### Characteristics of study participants

Table 1 presents the sociodemographic characteristics of the 50 study participants. The mean age of participants was 50.1 (SD = 16.6). More females (72%) than males participated. More than half (52%) of the participants had higher education level, 68% were employed, and 26% were retired. Approximately 20% had children under the age of 18, and 42% were married. Financial status was self-rated as good or fair by 90% of participants. 22% of the participants reported having long standing health problem lasting more than 6 month and 20% experienced limitations in usual activities due to health problems. Approximately half of the people lived in settlement located in rural areas, and half in a suburb of a city.

**Table 1:** Characteristics of study participants

			Previous gardening practice			
	Total	%	Yes	%	No	%
**Study participants**	50	100.0	35	70.0	15	30.0
Age* (mean,** SD)**	50.1	16.6	53.3	16.9	42.5	13.6
Sex*						
Male	14	28.0	6	17.1	8	53.3
Female	36	72.0	29	82.9	7	46.7
**Education level**						
High	26	52.0	17	48.6	9	60.0
Medium	22	44.0	16	45.7	6	40.0
Low	2	4.0	2	5.7	0	0
Employment status*						
Currently employed	34	68.0	19	54.3	15	100
Currently not employed	2	4.0	2	5.7	0	0
Student	1	2.0	1	2.9	0	0
Retired	13	26.0	13	37.1	0	0
**Marital status**						
Married	21	42.0	12	34.3	9	60.0
Single	12	24.0	10	28.6	2	13.3
Divorced or widowed	8	16.0	7	20.0	1	6.7
Living with partner	9	18.0	6	17.1	3	20.0
**Child under 18**						
Yes	10	20.0	6	60.0	4	40.0
**Perceived financial status**						
Good or fair	45	90.0	30	85.7	15	100
Bad	5	10.0	5	14.3	0	0
**Long standing health problem**						
Yes	13	22.0	10	76.9	3	23.1
**General activity limitation**						
Present	10	20.0	8	80.0	2	20.0
Settlement location*						
Rural	26	52.0	21	60.0	5	33.3
Suburb	24	48.0	14	40.0	10	66.7

Values are presented as mean (SD) for continuous variables and n (%) for categorical variables. Differences in the distribution of variables by previous gardening practice were statistically analyzed using independent samples t-test for age and Chi square test or fisher’s exact test for categorical variables. *Statistically significant differences are indicated by *p < 0.05

**Table 2:** Vegetable and fruit consumption, nutrition knowledge, physical activity, BMI and well being before and after the intervention in the total sample and by previous gardening status

Variables	Total (*n* = 50)		*p*- value*	Previous gardening (*n* = 35)		Never gardening (*n* = 15)		*p*-value**
	Before	After		Before	After	Before	After	
**Vegetable and fruit consumption**								
Servings/day (median, IQR)	1.6 (0.8–2.7)	5.5 (3.9–7.6)	< 0.001	1.9 (0.8–2.7)	5.9 (3.8–7.1)	1.1 (0.8–2.7)	5.4 (4.6–8.6)	0.676
Meet the recommendation (n, %)	5 (10.0)	30 (60.0)	< 0.001	4 (11.4)	21 (60.0)	1 (6.7)	9 (60.0)	1.000
**Vegetable consumption**								
Servings/day (median, IQR)	0.8 (0.4–1.7)	4.0 (2.6–6.6)	< 0.001	1 (0.4–1.7)	4.0 (2.6–6.6)	0.6 (0.4–1.1)	4.0 (2.6–8.6)	0.577
Meet the recommendation (n, %)	3 (6.0)	34 (68.0)	< 0.001	3 (8.6)	23 (65.7)	0 (0.0)	11 (73.3)	1.000
**Fruit consumption**								
Servings/day (median, IQR)	0.7 (0.3–1.3)	1.5 (1–2)	< 0.001	0.7 (0.3–1.3)	1.5 (1–2)	0.7 (0.3–1.3)	1.4 (1.1–2.1)	0.970
Meet the recommendation (n, %)	6 (12.0)	18 (36.0)	0.001	4 (11.4)	12 (34.3)	2 (13.3)	6 (40.0)	0.799
**Nutrition knowledge score** (median, IQR)	3.8 (3.4–4.5)	4.2 (3.8–4.6)	< 0.001	3.6 (3.2–4.2)	4.0 (3.6–4.8)	4.2 (3.4–4.6)	4.2 (3.8–4.8)	0.023
**Physical activity** (n, %) (physically active at least 150 min/week)	39 (78.0)	43 (86.0)	< 0.001	27 (77.1)	30 (85.7)	12 (80.0)	13 (86.6)	1.000
**BMI** (mean, SD)	26.5 (3.4)	26.1 (3.1)	0.523	26.3 (3.5)	25.9 (3.3)	27.3 (3.1)	26.6 (2.5)	0.234
**Well-being** (median, IQR)	8 (6–9)	8 (7–9)	< 0.001	8 (6–8)	8 (7–9)	8 (7–9)	8 (6–9)	0.869

*Differences in the distribution of variables before and after the intervention were statistically analyzed using Wilcoxon signed rank test for non-normally distributed variables (vegetable and fruit consumption, nutrition knowledge score, and well-being); paired t test were used for normally distributed variables (BMI); and McNemar test for categorical variables (meet the recommendations, physical activity)

**Differences in the changes in the measured variables between participants by previous gardening practice were statistically analyzed using Mann-Whitney U test for non-normally distributed variables (vegetable and fruit consumption, nutrition knowledge score, and well-being); the independent samples t-test for normally distributed variables (BMI); and Fisher’s exact test for categorical variables (meet the recommendations, physical activity)

A total of 70% of participants reported having gardening experience prior to the intervention. Participants with previous gardening experience were older, most of them were female, and their household were located in rural settlement. All participants with no prior gardening experience were currently employed. The average cultivated area was 102.1 m² (SD = 163.5). On average, those with gardening experience cultivated 4.4 types of plants prior to the intervention.

### Vegetable and fruit consumption

Vegetable and fruit consumption among participants increased significantly, increasing from a median of 1.6 servings per day (IQR: 0.8–2.7) before the intervention to 5.5 servings per day (IQR: 3.9–7.6) after the intervention.(*p* < 0.001). The proportion of participants meeting the WHO-recommended intake of at least 5 servings per day increased from 10% to 60% (*p* < 0.001). (Table 2)

Vegetable intake exhibited the greatest improvement, increasing from a median of 0.8 servings per day (IQR 0.4–1.7) to 4.0 servings per day (IQR 2.6–6.0) (*p* < 0.001). The proportion of participants meeting the recommended 2 servings of vegetable per day increased from 6% to 68% (*p* < 0.001). Fruit consumption also increased significantly, from a median of 0.7 servings per day (IQR 0.3–1.0) to 1.5 servings per day (IQR 1.0–2.0) across the total sample (*p* < 0.001). The proportion of participants meeting the recommended 3 servings of fruit per day increased from 12% to 36% (*p* = 0.001). (Table 2)

Comparisons between groups based on previous gardening experience revealed that participants with and without prior experience increased their consumption of fruits and vegetables. The increase in vegetable and fruit consumption was not significantly different between those who had previously gardened and those who had not.(Table 2)

### Healthy nutrition knowledge

The average healthy nutrition knowledge score increased from 3.8 (IQR 3.4–4.5) to 4.2 (IQR 3.8–4.6) (*p* < 0.001). Scores range from 1 to 5, with 1 indicating lower knowledge and 5 indicating higher knowledge. Participants with previous gardening experience exhibited a slightly greater increase in scores than those without prior experience (*p* = 0.023). (Table 2)

### Physical activity

The proportion of participants meeting the physical activity guidelines increased from 78% to 86% after the intervention (*p* < 0.001). There were no differences between the two groups in terms of previous gardening experience.(Table 2)

### Body mass index (BMI)

The mean BMI decreased slightly from 26.5 (SD = 3.4) to 26.1 (SD = 3.1), but this change was not statistically significant (*p* = 0.523). There were also no differences between the two groups in terms of previous gardening experience. (Table 2)

### Well-being

There was a slight change in the distribution of well-being scores (life satisfaction on a 1–10 scale) from a median of 8 (IQR 6–9) to 8 (IQR 7–9) after the intervention. (*p* < 0.001). There were no differences between the two groups in terms of previous gardening experience. (Table 2)

## Discussion

The results of this study suggest that the home gardening intervention had a positive effect on daily vegetable and fruit intake of study participants. The proportion of participants who met the recommended daily intake of 5 portions increased from 10% to 60%. The daily intake of vegetables increased substantially from a median of 0.8 servings (IQR 0.4–1.7) to 4.0 servings (IQR 2.6–6.0). The substantial improvement in vegetable intake aligned with the intervention design, participants received seedlings and seeds, as well as support through educational sessions. Although we only provided vegetable seeds and seedlings, fruit intake also increased significantly, what can be attributed to the complex nature of the intervention. The increase in fruit consumption is likely due to nutrition education, increased general awareness of dietary recommendations and greater motivation to eat healthily. These mechanisms may have been reinforced by the group sessions, practical guidance and ongoing communication via social media, encouraging participants to make broader improvements beyond increasing their vegetable intake. Furthermore, immediate feedback was provided by the visible results of home gardening efforts, i.e. harvested vegetables, which boosted self-esteem, pride and life satisfaction [20]. Behavioral theories emphasize that knowledge, self-efficacy, and environmental support interact to facilitate the modifications in eating habits [21].

Our findings are consistent with previous studies showing that relatively short-term (e.g., three months) home gardening interventions can meaningfully increase vegetable and fruits consumption [16, 22]. Moreover, access to a home garden was itself associated with higher baseline intake and healthier eating habits [23].

Many prior studies in low- and middle-income countries have shown similar benefits, primarily through improved household food security and dietary diversity [24–29]. Although the context differs, the underlying mechanisms - expanded access to fresh produce, enhanced food skills, improved dietary literacy, and strengthened health motivation - appear to be shared across populations. In addition, the slight increase in physical activity observed in our study may relate to the physically engaging nature of gardening, which has been documented in earlier research showing that routine gardening tasks can contribute meaningfully to moderate-intensity activity levels [30, 31]. Although, we were unable to demonstrate improvements in BMI, in contrast to the findings of other studies [32, 33]. This can be attributed to the relatively short follow-up period of the study.

The Hungarian context further highlights the relevance of our findings. In Hungary, poor diet accounts for the largest part of mortality, followed by tobacco smoking according to the OECD State of Health in the EU reported in 2021. One-quarter of all deaths in 2019 could be attributed to dietary risks, which is far above the EU average of 17% [34]. National dietary survey data indicated very low levels of fruit and vegetable consumption in Hungary, only 8.2% of people reported to consume the recommended daily 5 portions of fruits and vegetables and 36.3% of respondents reported not consuming any portion of fruits and vegetables daily [35]. Considering this situation, there is an urgent need for interventions to improve diet including increasing the consumption of fruits and vegetables. Our study demonstrated that home gardening interventions can have a positive effect on the intake of fruits and vegetables and are potentially a feasible way of promoting healthier eating habits in rural communities.

The strength of this study is its comprehensive approach, which combines financial support (in the form of educational tools, seeds and seedlings) with structured education and monitoring, to promote meaningful behavioral change. The intervention was practical and low-cost, and was tailored to a rural population, thereby enhancing its real-world applicability. The study evaluated various aspects of health, including diet, physical activity, knowledge and well-being, in line with public health frameworks. The absence of loss to follow-up strengthens the internal validity of the findings. However, several limitations must be acknowledged. The interventional study lacked a control group, limiting the ability to attribute observed changes exclusively to the intervention, therefore causality cannot be determined. Additionally, the reliance on self-reported data introduces the possibility of social desirability bias and recall bias. Dietary intake was assessed with a semiquantitative food frequency questionnaire which is less accurate than 3-day dietary record. Seasonal variations in vegetable and fruit intake can also lead to bias. Additionally, it was not possible to ascertain whether the observed increase in vegetable intake derived from home-produced or purchased sources, as such information was not collected. Similarly, the specific types of vegetables consumed in greater quantities could not be identified. Furthermore, the sample size was relatively small and volunteer bias likely occurred. Study participants were recruited from only two rural settlements which limits the generalizability of the findings. The intervention duration of 3.5 months may not have been sufficient to detect longer-term outcomes, such as changes in BMI or long-lasting dietary improvements. Despite these limitations, however, this study provides valuable insights into the potential of home gardening programs to improve dietary habits and health-related behaviors in underserved rural populations.

## Conclusion

The findings of this study suggest that home gardening interventions can increase the consumption of fruits and vegetables among rural populations in Hungary. The study was based on the implementation of a low-cost intervention at community-level. The results suggest that home gardening is a feasible public health strategy in disadvantaged rural areas. Larger scale studies should assess whether home gardening can induce long-term dietary changes and explore its integration with community-based public health programs.

## Data Availability

Data supporting the findings is available at reasonable request from the corresponding author.

## Abbreviations

VFs: Vegetables and fruits
BMI: Body mass index
SD: Standard deviation
IQR: Interquartile range
PA: Physical activity
